# Immune cell type signature discovery and random forest classification for analysis of single cell gene expression datasets

**DOI:** 10.3389/fimmu.2023.1194745

**Published:** 2023-08-04

**Authors:** Bogac Aybey, Sheng Zhao, Benedikt Brors, Eike Staub

**Affiliations:** ^1^Oncology Data Science, Merck Healthcare KGaA, Darmstadt, Germany; ^2^Faculty of Biosciences, Heidelberg University, Heidelberg, Germany; ^3^Division of Applied Bioinformatics, German Cancer Research Center, Heidelberg, Germany; ^4^German Cancer Consortium, German Cancer Research Center, Heidelberg, Germany

**Keywords:** single-cell RNA sequencing, gene signature discovery, cell type classification, machine learning, cell clustering, tumor microenvironment

## Abstract

**Background:**

Robust immune cell gene expression signatures are central to the analysis of single cell studies. Nearly all known sets of immune cell signatures have been derived by making use of only single gene expression datasets. Utilizing the power of multiple integrated datasets could lead to high-quality immune cell signatures which could be used as superior inputs to machine learning-based cell type classification approaches.

**Results:**

We established a novel workflow for the discovery of immune cell type signatures based primarily on gene-versus-gene expression similarity. It leverages multiple datasets, here seven single cell expression datasets from six different cancer types and resulted in eleven immune cell type-specific gene expression signatures. We used these to train random forest classifiers for immune cell type assignment for single-cell RNA-seq datasets. We obtained similar or better prediction results compared to commonly used methods for cell type assignment in independent benchmarking datasets. Our gene signature set yields higher prediction scores than other published immune cell type gene sets in random forest-based cell type classification. We further demonstrate how our approach helps to avoid bias in downstream statistical analyses by re-analysis of a published IFN stimulation experiment.

**Discussion and conclusion:**

We demonstrated the quality of our immune cell signatures and their strong performance in a random forest-based cell typing approach. We argue that classifying cells based on our comparably slim sets of genes accompanied by a random forest-based approach not only matches or outperforms widely used published approaches. It also facilitates unbiased downstream statistical analyses of differential gene expression between cell types for significantly more genes compared to previous cell classification algorithms.

## Introduction

Recent improvements in single cell technologies led to a multitude of singe cell RNA-sequencing (scRNA-seq) studies to understand the complex interplay of immune cells in human tissues (1). The procedures to assign cell types to single cells in such data are a critical factor for the success of such studies (2). There are hardly any ‘golden rules’ or generally accepted computational workflows for assigning cell type labels to cells. Still, cell types are often annotated manually: after unsupervised clustering of all cells a manual assignment of cell types to clusters is performed by assessing expression patterns of author-selected marker genes (3, 4). Manual cell type assignment after assessment of small sets of markers is error-prone: it can depend on habits and non-explicit rules and opinions of researchers. Both steps are major sources of irreproducibility in the field (5). Many researchers demonstrate that larger sets of genes, gene signatures with sometimes dozens of genes, can provide more reliable information for cell type classification since e.g., not all cells express even the most widely used literature marker genes of their corresponding cell types (6). Therefore, different sets of immune cell type gene expression signatures have been proposed (7–11). Most of these gene sets have been derived by analyses of bulk tissue-based RNA-sequencing (RNA-seq) datasets, the older studies have used microarray-derived gene expression data. Most recent signature sets have been derived by analysis of scRNA-seq data (12–14). However, only single gene expression experiments have been analyzed to derive these signature sets. Although it is obvious that utilization of multiple datasets generated by different labs or technologies could enable the identification of more robust gene signatures, immune cell type gene signatures based on the integration of evidence from multiple published scRNA-seq gene expression studies have not been described so far.

There is a specific group of workflows for cell type annotation and downstream analysis that depends on clustering the cells in one of the initial steps of cell type labeling (15, 16). Such cell type annotation processes are often strongly affected by clustering results which heavily depend on the clustering parameters (4). Clustering and cell type annotation heavily affect downstream analysis. When clustering and differential gene expression testing has used information from all genes for cell typing this leads to statistical biases (2). It is obvious that pre-requisites for statistical analyses are violated when first information from all gene profiles is used for establishing groups of cells, and later these same gene expression data and derived cell groups are used for statistical tests to determine differential gene expression between the same groups (5). Similarly, a violation of statistical independence can be observed in most automatic cell type annotation approaches which make use of a complete reference dataset plus the data to be annotated. The problem arises when the focus is set on highly variable genes in both, the reference, and new dataset, instead of using only the reference data as information source about gene variance: the new data is already used for training of the classification model (17). This method of cell type classification maybe is not problematic in general, but it becomes problematic when researchers are interested in the investigation of differential expression of HVGs between cell types by statistical testing. Such bias should ideally be avoided or reduced in all workflows which use cell type annotation followed by investigation of differences between cell groups. Automated deterministic approaches that lead to reproducible cell type annotations would not only be beneficial for the interpretation of single studies, but they would also facilitate cross-comparison between single cell gene expression studies. Pre-trained machine learning models that make use of only expression information of a small fraction of the genes and leave the majority of gene expression information untouched for unbiased downstream statistical analyses could be a significant step forward.

In our study, we established a novel discovery workflow to identify immune cell-specific gene expression signatures by leveraging multiple scRNA-seq tumor microenvironment datasets. Furthermore, to eliminate the sources of analytical bias and increase reproducibility, we developed a random forest-based cell type classification approach operating on small sets of genes of our immune cell type signatures. We extensively tested the performance of our gene sets and our classification approach against other widely used cell annotation approaches on two peripheral blood mononuclear benchmarking datasets.

## Data and methods

### Single cell RNA-seq datasets and quality control for genes and cells

We list all datasets used in this study for expression signature discovery, validation, classifier training and benchmarking purposes in **Table 1**, along with quality control metrics and dataset information. For all discovery datasets, we included only those immune cell types from tumor microenvironments in our analyses which were present in at least three discovery datasets. We obtained log-normalized expression matrices from the TISCH2 database (29) for the datasets that we used for discovery of signatures. As a validation dataset, we used the tumor immune cell atlas (24). We removed all samples from datasets that we had used for discovery purposes from the Nieto cell atlas that we used for validation.

**Table 1:** List of datasets used in this study along with their quality control measures.

Dataset	Source	Cell source	Sequencing technology	Purpose	Number of cells, average number of genes per cell	Portion of mitochondrial genes [<%]	Unique gene count
GSE176078	(18)	BRCA	10X	Discovery	43,140 12,661	5	<10,000
GSE166555	(19)	CRC	10X		13,369 12,681		
GSE140228	(20)	LIHC	Smart-Seq2		2,351 39,531		
GSE140228	(20)	LIHC	10X		16,724 13,751		
GSE139555	(21)	KIRC	10X		18,120 11,861		
GSE131907	(22)	NSCLC	10X		25,915 14,051		
GSE123139	(23)	SKCM	MARS-Seq		4,817 8,861		
TIC Atlas	(24)	13 cancer types	Various	Validation	229,753 1,265	15	<5,000
Hao	(25)	PBMC	CITE-seq	Reference	158,783 2,207	15	>500, <6,000
Kotliarov	(26)	PBMC	CITE-seq	Benchmarking	52,849 748	10	<2,500
Zheng	(27)	PBMC	10X		18,000 562	5	<1,500
Kartha	(28)	PBMC	SureCell Biorad	Differential gene expression (investiagtion of bias)	23,754 943	–	<4,000

For all datasets, we removed cells expressing less than 200 genes and genes expressed in less than three cells and normalized the count matrices using ‘LogNormalize’ by *Seurat* (v4.3.0) (16). We used the cell type annotations published by the original authors to annotate and validate our expression signatures and to benchmark cell type classification methods. To investigate the statistical bias when cell type classification uses the same genes that are later subjected to statistical testing, we used the dataset of Kartha et al. (28): we only included type II interferon treatment and control samples for this purpose.

### Gene sets for comparison to our approach

In addition to our own immune cell signatures, we investigated the following public immune cell signature repertoires: Abbas (7), Charoentong (11), Angelova (9), Becht (30), Bindea (8), Newman (31), Nirmal (32) and Nieto (24). For comparing our gene signature collection with other gene set collections in a random forest approach, we focused on the following studies and cell types: a) Abbas: B cells, DCs, monocytes, NK, and T cells; b) Charoentong: B cells (general and memory), DCs (immature and pDC), monocytes, NK and CD4^+^ (regulatory, effector memory, central memory, and general) and CD8^+^ T cells (effector memory, central memory, and general); c) Angelova: B cells (memory and immature), DCs (pDC, immature, mDC and general), monocytes, NK, and CD4^+^ (regulatory, effector memory, and central memory) and CD8^+^ T cells (effector memory and central memory); d) Nieto: B and plasma cells, DCs (mDC, cDC and pDC), monocytes, NK cells, naïve T cells, CD4^+^ T (effector memory, transitional memory, memory/naïve and regulatory) and CD8^+^ T (effector memory and cytotoxic) cells.

### Dataset integration

For the integration of multiple scRNA-seq datasets we used reciprocal principal component analysis (RPCA)-based integration implemented in *Seurat* (v4.3.0) (16). To find anchors between discovery datasets and to integrate the datasets, we used Seurat standard functions (https://satijalab.org/seurat/articles/integration_rpca.html).

### Dimension reduction and spatial clustering

To cluster genes with similar expression profiles in our integrated expression matrix, we used a density-based clustering approach. Prior to clustering, we reduced the dimensionality of the Z-scaled integrated expression matrix using UMAP from *uwot* (v0.1.14) (33) on the gene dimension to the first and second UMAP components. On this spatial representation of the UMAP space -in which each point represents a gene- we performed density-based clustering using *dbscan* (v1.1-11) (34), thereby clustering genes into gene clusters. *dbscan* operated with two parameters: minimum points (minPts) in a gene cluster and maximum distance between two data points (epsilon).

### Gene cluster refinement using silhouette scores and mean signature scores

The silhouette scores were used to evaluate the quality of the gene elements and refine the gene clusters by considering the similarity of gene expression profiles within clusters and the dissimilarity between clusters. We calculated silhouette scores for individual genes and clusters using *cluster* (v2.1.1) (35). As inputs we used the cluster labels from *dbscan* and the gene-by-gene correlation distance matrix for genes x and y: d_x,y_ = 1 - *r*(x,y), where *r*(x,y) represents the Pearson correlation calculated from Z-scaled integrated expression profiles of genes x and y. The silhouette scores range from -1 to +1, with higher values indicating better clustering results and values closer to negative suggest that the sample is likely to be assigned to the wrong cluster.

To evaluate the expression strength of each signature in each cell type, we employed the “Average Z-Score method.” This method allows us to measure the relative expression level of a signature in a cell by considering the expression values of all genes associated with that signature. We averaged the Z-scaled expression values (mean-centered and standardized across cells) for each gene within the signature in each cell to obtain mean signature score for a cell. Subsequently we represented average mean signature scores for each cell type by averaging mean signature scores coming from the cells belonging to a given cell type.

### Quantifying gene set similarities

We measured the similarity of the gene sets (overlap of sets) by calculating the Jaccard index using *bayesbio* (v1.0.0) (36) and the Szymkiewicz–Simpson coefficient (37) between ours and all published signatures. The Jaccard index calculates the ratio of the intersection of two sets (our gene set and a published gene set) to the union of both sets. It provides a measure of the proportion of shared genes between the sets, indicating the degree of similarity. Similarly, the Szymkiewicz–Simpson coefficient also quantifies the similarity between two sets by dividing the intersection of two sets by the number of elements belonging to the set with minimum number of elements. This coefficient provides an alternative measure to assess the overlap between gene sets considering the differences between set sizes and evaluate the similarity of the genes identified in our study with those reported in the literature.

### Cell type classification and performance benchmarking of cell type classification tools

For our immune cell type classification approach, we applied our immune cell type gene signatures as features in a random forest approach. For building random forest models utilizing *randomForest* (v4.6-14) (38) we used a ratio of 67:33 for random sampling of training and test data. We used only common signature genes between reference and query datasets as features. Prior to the training, we harmonized original cell type annotations from training datasets at medium-depth level shown in **Supplementary Table 1** and only included medium level cell types (monocytes, DCs, B, NK, CD4^+^ T and CD8^+^ T cells). For the assessment of the performance of other published gene signatures, we applied an analogous procedure. Further, we used five different cell type annotation tools with default parameters: *Seurat* (v4.3.0) (16), *singleR* (v1.4.1) (15), *scType* (39), *CellTypist* (v1.5.0) (40) and *CHETAH* (v1.6.0) (41). Prior to the cell type prediction using Seurat, we applied the standard pipeline to the query and reference dataset including log-normalization, finding and scaling HVGs. The anchors for cell type label transfer were determined between reference and query datasets and cell type labels were then transferred to the query dataset based on the PCA projection. For singleR and CHETAH, predictions were obtained by providing normalized query and reference dataset along with cell type labels from a reference dataset. For CellTypist, we utilized the same reference dataset to train the model and used the default settings with majority voting option. As input expression matrix, we used the raw expression values for the reference and query datasets. In the case of scType, it differs from other algorithms as it does not rely on any reference dataset to label cells. Instead, it utilizes information from cell clusters and specific combinations of cell type markers. We followed the standard workflow of normalization, scaling, and clustering using *Seurat*. Additionally, we loaded gene sets from the built-in ‘Immune system’ database provided by scType. To assign cell types to clusters, we followed the suggested steps recommended by scType. In the case of perturbation datasets, we provided RPCA-based integrated matrix as input to Seurat and singleR as suggested by the methods.

Prior to the predictions, we harmonized original cell type annotations from benchmarking datasets at medium-depth level shown in **Supplementary Table 1**. To evaluate the performance of the cell type prediction algorithms, we used six statistical metrics: accuracy, specificity, sensitivity, negative predictive value (NPV), positive predictive value (PPV) and F1-score. We reported the mean of each statistical metric for each algorithm.

## Results

Our study is structured into three parts: First, we comprehensively analyzed gene expression similarities across different single cell expression datasets of tumor microenvironments to identify immune cell signatures. We discovered and refined gene modules to finally obtain robust gene signatures for immune cell types. Second, we tested the utility of our gene sets as classification features in a random forest-based (RF) immune cell type classification approach. We compared our approach with five most widely used methods on two independent benchmarking datasets. Third, we examined how our gene sets are compared to other published gene signature sets or alternative feature selection approaches, and how the way of choosing genes as features for classification influences the performance of a RF-based classification approach for cell type annotation.

### Discovery of robust immune cell type gene expression signatures by leveraging multiple scRNA-seq datasets

**Figure 1:**
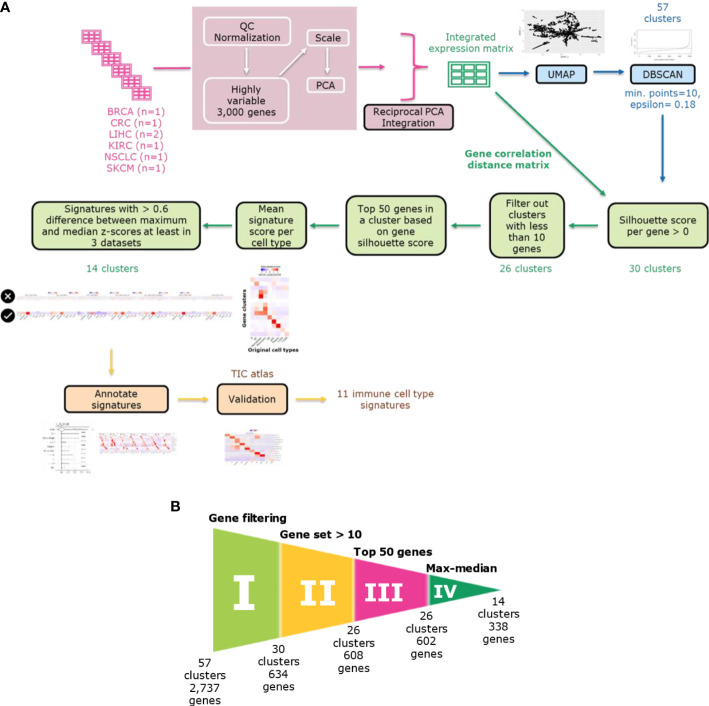
Immune cell type gene signature discovery workflow. **(A)** The workflow comprises the following steps: dataset integration, density-based clustering using DBSCAN, refinement of gene sets using filtering approaches based on silhouette scores and mean signature expression score and annotating and validating the signatures. **(B)** Funnel plot showing the refinement process in each step. The refinement process consists of five filtering steps: gene filtering based on silhouette scores, selection of gene sets with minimum ten genes, selection of top 50 genes based on silhouette scores and max-median filter based on mean signature expression scores. Each step is labeled from I to IV. The final number of clusters and genes is shown after each filtering step.

**Figure 2:**
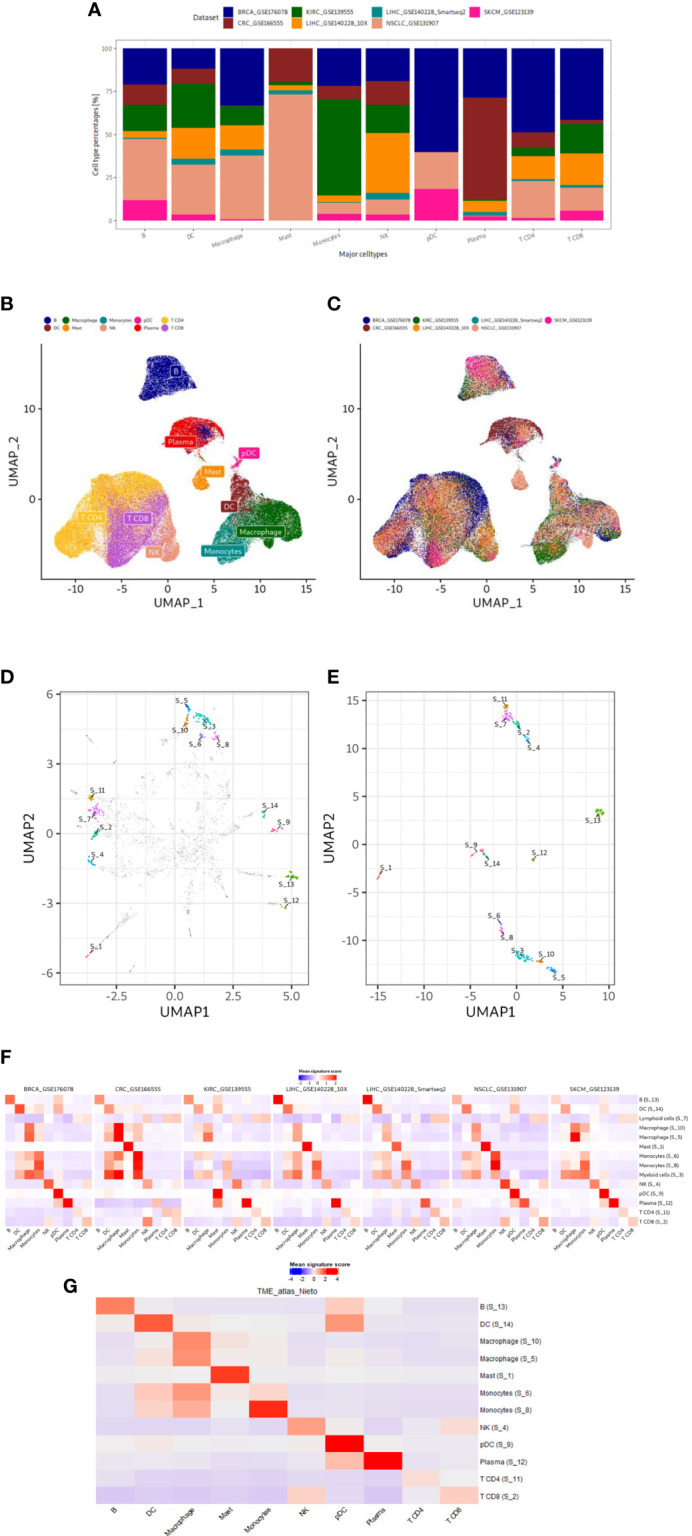
Data characteristics and results of our gene signature discovery. **(A)** Contribution of discovery datasets to each immune cell type. **(B, C)** UMAP plots of integrated expression matrix. Each point represents a single cell, and each cell is colored by cell type **(B)** or dataset **(C)**. The cell type labels are taken from the original publications. Cell type labels are placed in the center of the cell type clusters. Note the successful integration and harmonization of the datasets. DC, dendritic cell; NK, natural killer; pDC, plasmacytoid dendritic cell. **(D, E)** UMAP plots based on 3 HVGs or final genes from our gene signatures. The dimensionality of the gene space of expression data is reduced in each step, starting from 3k common HVGs in the integration step to finally 338 genes of our gene sets. Each point represents a gene. UMAP1 and UMAP2 are plotted for each gene in x and y axis, respectively. In D and E genes in our gene signatures are annotated in different colors. In D other genes are colored gray. Cluster numbers are placed in the center of the clusters. Genes from each refined gene set cluster together to the exclusion of other gene sets. **(F, G)** Mean signature expression scores per cell type of refined gene signatures shown in the discovery and validation datasets. Red and blue represent high and low mean signature expression scores, respectively. Rows represent the gene signature cluster numbers along with the manual annotations while columns represent the cell types defined by the original authors in the datasets. The signature annotation names contain cell type which the signature can detect. Discovery and validation datasets are shown in F and G, respectively.

To discover immune cell signatures, we established an integrated density-based clustering workflow leveraging multiple scRNA-seq datasets (**Figure 1**). We integrated seven tumor immune microenvironment scRNA-seq datasets of treatment-naïve tumors from six cancer types: skin cutaneous melanoma SKCM, liver hepatocellular carcinoma LIHC, breast cancer BRCA, kidney renal clear cell carcinoma KIRC, non-small cell lung cancer NSCLC and colorectal cancer CRC. Each discovery dataset contributed differently to the ten medium level immune cell types (**Figure 2A**). We applied reciprocal principal component-analysis (RPCA) based integration based on top 3k highly variable genes (HVGs) and analyzed the successfully integrated expression matrix in a UMAP analysis: different studies were harmonized in different immune cell type clusters and similar cell types with similar expression profiles clustered together (**Figures 2B, C**). The integrated gene expression matrix comprised expression profiles of 3k genes for 123,509 cells from seven datasets and subsequently served us to discover immune cell type-specific gene signatures.

To identify clusters of genes with similar expression profiles in our integrated dataset, we utilized a density-based clustering approach (**Figure 1**). We reduced the dimensions of the Z-scaled integrated data in the gene space -not the cell space as used for the integration- to the first and second components of UMAP. We applied a spatial clustering approach, the DBSCAN algorithm, on the UMAP space, where each data point corresponds to a gene, to cluster genes into gene clusters. To determine two DBSCAN clustering parameters (epsilon and minimum number of points in a cluster minPts), we examined the optimal epsilon after plotting k-nearest neighbor distances in ascending order and analyzing the ‘knee’ point where maximum curvature was observed for a minimum of ten genes (minPts) in a gene cluster. The optimal epsilon was at 0.3 but since we aimed to obtain more clusters and have a higher resolution, we considered lower epsilon values as our epsilon candidates. For the selection of the epsilon value which captures signatures for all immune cell types, we tried different values ranging from 0.15 and 0.2 in 0.05 interval. To do so, we ran the gene refinement workflow (**Figure 1**) explained in the following parts including filtering out genes with negative silhouette scores and taking only top 50 genes with the highest silhouette scores. Then, in the given range, we examined the heatmaps showing the mean signature scores - as calculated using the Average Z-Score method- for each cell type in each discovery dataset. An epsilon of 0.18 resulted in a better resolution capturing signatures for all cell types in the discovery datasets. Subsequently, applying DBSCAN with given parameters (epsilon=0.18 and minPts=10) on the UMAP gene space we obtained 57 gene clusters, each with a minimum of ten genes.

To refine the gene content within each cluster, we employed silhouette scores (**Figure 1B** I). These served as an evaluation metric for assessing the quality of gene clusters and individual gene elements. We calculated the silhouette scores for genes based on the gene-by-gene correlation distance matrix. We filtered out genes which did not align well with their respective clusters, specifically those with negative silhouette scores. Further, we filtered out clusters with less than ten genes. For all clusters, we only included the top 50 genes in each cluster based on the gene-wise silhouette scores (**Figure 1B** II-II). We assessed how strong the mean relative gene expression score (averaged Z scores of all genes in cluster) is in all cell types as they had been annotated in the original publications. We noticed that some gene clusters might not define cell types but rather biological programs (e.g., IFN response and cell cycle) and decided to not further focus on such gene sets. To filter out those gene sets, we removed clusters when they exhibited only a small difference (<0.6 Z score units on natural log scale) between the cell type with maximum expression score and the cell type with median score in at least three discovery datasets (**Figure 1B** IV). We finally assessed the success of our gene selection strategy by performing an UMAP dimension reduction focusing only on the genes from our 14 gene clusters (**Figures 2D, E**). On the initial UMAP used for clustering (**Figure 2D**), genes within each of those 14 gene sets were forming distinct clusters that were separate to the exclusion of genes not belonging to the gene set. We obtained a congruent clustering pattern when we re-applied UMAP on the Z-scaled expression of only those 338 genes from our 14 gene sets (**Figure 2E**). These findings show the recurrent segregation of genes into the same clusters in early and late phases of our gene signature discovery process.

After gene set refinement, we finally obtained 14 gene sets that were subjected to a comprehensive annotation and validation approach. To annotate the signatures, we examined mean gene expression scores across all cell types (as identified by original authors) in each discovery dataset (**Figure 2F**). We manually annotated the gene sets based on which immune cell type had the highest expression of a given signature at least in three discovery datasets in the depicted heatmap: B, DC, macrophages, mast, monocytes, NK, pDC, plasma and CD4^+^ and CD8^+^ T cells. We also obtained two lineage signatures: myeloid and T cell lineages. To statistically validate our manual annotations, we tested the enrichment of each signature in its corresponding cell type using Wilcoxon rank sum test comparing signature scores of all cells of a given cell type against cells from other cell types (**Supplementary Figure 1**). All of 14 signatures were higher expressed in their cell types (uncorrected p<0.01) in at least three discovery datasets. Since we have aimed to discover immune cell type signatures for unique cell types, from now on we will only focus only on the twelve immune cell type signatures and not on the two immune cell lineage signatures.

To validate our twelve gene sets and prove the correctness of our immune cell type annotations, we used the scRNA-seq gene expression dataset from tumor immune cells atlas (24) as a validation dataset: it has not been used for gene signature discovery. We established mean signature expression scores for each cell and assessed these across cell types mentioned in the original study; we also tested statistical enrichment of the signatures in each cell type (**Figure 2G**; **Supplementary Figure 1**). Finally, we could validate all our signatures except the S_6 monocyte signature. We summarize our final validated gene list of eleven immune cell type gene signatures in **Table 2**.

**Table 2:** Summary of our refined immune cell type signatures.

Cluster number	Cluster annotation	Genes	Number of genes
S_13	B	TCL1A, VPREB3, CD22, EBF1, FCER2, STAG3, MS4A1, PARP15, CD79B, KHDRBS2, BANK1, FAM129C, CD79A, CXCR5, LINC00926, BACH2, AFF3, LY9, RALGPS2, SMIM14, FCRLA, CD37, SPIB, FCRL1, IRF8, CD19, CNR2, TNFRSF13B, ADAM28, COL19A1, PAX5, ARHGAP24, TCF4, BLK, PKIG, RIC3, IFT57, TNFRSF13C	38
S_14	DC	CD1E, HLA-DQB2, CD1B, PKIB, CALCRL, CD1A, FCER1A, S100B, PLD4, CD1C, PPP1R14A, NAPSA, CD207	13
S_10	Macrophage	APOE, CTSL, GPNMB, CD9, TREM2, CTSD, APOC1, ADAMDEC1, SPP1, MMP9, PLA2G7, LIPA, ACP5, NUPR1, FN1	15
S_5	Macrophage	CCL13, MS4A4A, SLC40A1, LYVE1, RNASE1, SIGLEC1, C1QA, STAB1, CXCL12, ABCA1, IGF1, GPR34, PLTP, C1QB, PMP22, A2M, LGMN, FOLR2, SLCO2B1, MRC1, DAB2, NRP1, LILRB5, C1QC, F13A1, PLAU	26
S_1	Mast	TPSAB1, HPGDS, ADCYAP1, CPA3, PLAT, GATA2, CTSG, HPGD, KIT, CLU, IL1RL1, KIAA1549, RSPH9, SYTL4, HDC, VWA5A, RGS13, TPSB2, LIPC, SLC18A2	20
S_8	Monocytes	LILRA5, SLC25A37, CFP, S100A12, CD300E, TIMP1, APOBEC3A, FCN1, TREM1, SLC11A1, VCAN, S100A9, S100A8, CDA, THBS1, FGR	16
S_4	NK	GZMB, CD160, TXK, KIR2DL4, TMIGD2, CTSW, KRT86, KLRF1, SH2D1B, GNLY, PRF1, KLRD1, XCL2, CLIC3, XCL1, HOPX, MATK, PTGDR, KRT81, KLRC1	20
S_9	pDC	SCT, RGS7, IRF4, VASH2, GPM6B, MAP1A, NME8, PTCRA, PTGDS, AEBP1, CLEC4C, SMPD3, TTC39A, PHEX, MMP23B, PLVAP, PLAC8, RASD1, LILRA4, PTPRS, DNASE1L3, LRRC26, SLC35F3, TPM2, KRT5, TSPAN13	26
S_12	Plasma	IGLL5, FKBP11, ITM2C, XBP1, DPEP1, SEC11C, HSP90B1, TNFRSF17, SDC1, CAV1, SSR4, DERL3, MZB1, JSRP1, CERCAM	15
S_11	T CD4	FAS, TNFRSF25, PBX4, FAAH2, ICOS, CD28, CCR4, TMEM173, MAL, LTB, ARID5B, PBXIP1, TNIK, NPDC1, LEF1, FBLN7	16
S_2	T CD8	FASLG, CCL5, RAB27A, CD8B, CPNE7, CST7, OASL, GZMH, GZMA, CHST12, SAMD3, CLEC2B, CD8A, APOBEC3G, GZMM, SLA2, TNIP3, IFNG, TSEN54, CRTAM, C12orf75, LAG3, GZMK	23

*DC, dendritic cell; NK, natural killer cell; pDC, plasmacytoid dendritic cell.

It is possible that with our approach we just re-discovered gene sets that were already known. Thus, we compared our eleven immune cell type signatures with seven published immune cell signature repertoires using the Jaccard and Szymkiewicz-Simpson (S-S) indices. Our immune cell signatures had relatively low maximum Jaccard indices (0-0.32, median 0.12) (**Supplementary Figure 2A**). Four of our immune cell signatures (plasma, pDC, monocytes and macrophages) had maximum Jaccard indices below 0.1 suggesting that they are potentially novel signatures. Three highest detected Jaccard indices were found between our mast cell signature and Bindea mast signature, our B cell signature and the Nirmal and Newman B cell signatures (Jaccard idx= 0.32, 0.31 and 0.21, respectively. Maximum S-S indices had higher scores and varied in a range from 0.08 to 0.67 (**Supplementary Figure 2B**). The maximum three S-S- indices were between our B cell and Becht B cell signature (SSidx = 0.67), between our mast and Bindea mast signature (Ssidx = 0.6), between our B signature and Nirmal B cell signature Ssidx = 0.5). This shows that we have detected largely novel gene sets with significant differences in their composition compared to already published signatures, and that our signature gene sets were nearly always smaller than other gene sets.

In summary, we obtained eleven gene signatures for ten distinct immune cell type populations that exhibit high gene expression signature coherence in our seven discovery datasets and in one validation dataset.

### Using a limited number of cell type-specific genes combined with a random forest approach yields higher or comparable prediction scores in comparison to commonly used algorithms

**Figure 3:**
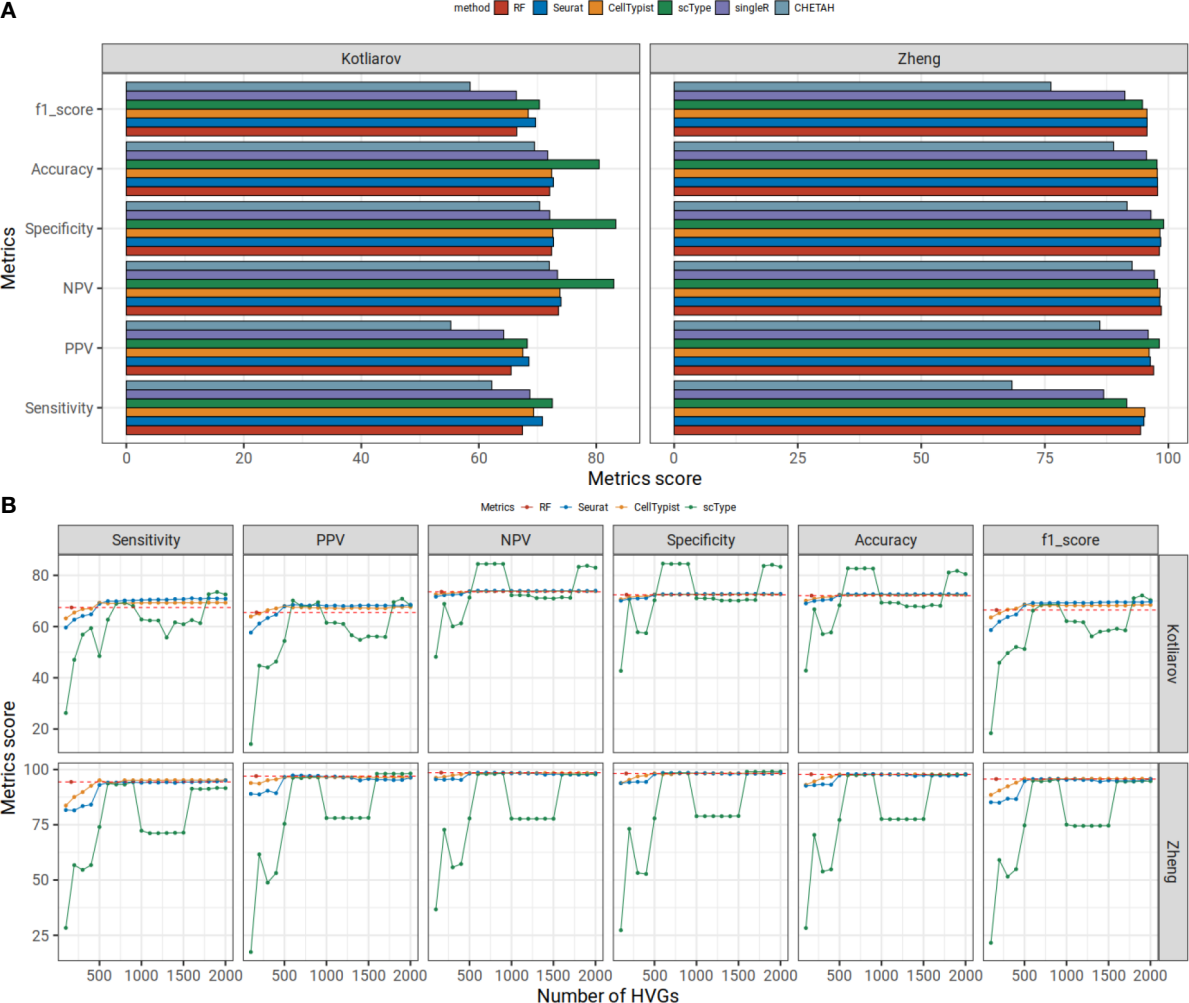
**(A)** Our random forest model shows higher or comparable prediction statistics compared to five commonly used tools in benchmarking datasets. Mean statistic metrics are displayed for each method. The Hao dataset is used as a reference dataset. The Kotliarov and Zheng datasets are used for benchmarking. **(B)** Prediction metrics change with increasing number of HVGs. In an interval of 100 HVGs, we predicted the cell type labels executing Seurat, CellTypist and scType using Hao reference data on Kotliarov or Zheng benchmarking datasets. We report the mean scores for six statistical metrics for each HVG set. The prediction scores for our RF approach are shown in red points and dashed lines.

Most cell type annotation approaches use information from a very large number of HVGs (often 2k genes are used) for cell typing. This might lead to problematic bias in down-stream statistical analyses of differential gene expression between cell types because most important genes have been already used up for cell typing (2). We hypothesized that using a small set of robust cell type signatures we can predict cell types as well as or better than approaches that use many more genes for cell typing and can therefore eliminate biases in the analyses of those many genes. We applied our immune cell type genes from our discovery workflow on a random forest cell type classification model (RF) and predicted cell types from two PBMC benchmarking datasets. As our gene features, we utilized 167 genes from our signatures for eight different cell type populations (plasma, monocytes, DC, pDC, B, NK, CD4^+^ and CD8^+^ T cells). Here we did not include genes from our two macrophage signatures and one mast cell signature since these were not suitable for PBMC data and we aimed to compare later our signature genes with published signatures of the same or similar signature content. For the training and prediction, we used the Z-scaled expression values only from the common signature genes between training and benchmarking datasets. As training data, we used a PBMC reference dataset- Hao dataset (25). We used medium-level cell types shown in **Supplementary Figure 1**. We trained RF using randomly selected 67% of reference data and tested on the left-out (33%) reference data. We compared our predictions with the most widely used cell typing tools, Seurat singleR, CHETEAH, scType and CellTypist on the Kotliarov PBMC benchmarking CITE-seq dataset. Cell type annotations in the Kotliarov dataset are based on cell surface marker protein expression which until now still is considered to be the gold standard for assigning cell types by many researchers (26). For Seurat and scType, we provided 2k HVGs as input while for CHETAH, singleR and CellTypist, the input contained all genes. First, we trained the models using Hao PBMC reference dataset for all methods except scType since scType relies only on the query dataset. scType (scores range in 69-83) and Seurat (scores range in 68-74) used with 2k HVGs yielded the highest prediction scores followed by CellTypist (scores range in 67-74) and our RF approach which had medium-high prediction scores (scores range in 65-74) (**Figure 3A**). Of note, relatively higher performance of scType is due to not considering unconventional T cells and hemopoietic stem cells in our reference data. CHETAH (scores range in 59-72) and singleR (scores range in 64-73) had the lowest predictions scores and singleR had lower or comparable benchmarking results compared to our RF approach except in sensitivity. CHETAH had lower prediction scores than RF in each metric.

We further examined the effect of gene choice for those algorithms which had slightly higher or comparable scores than our RF model: CellTypist, Seurat and scType. We applied these approaches using different numbers of HVGs. When using only small numbers of HVGs (<500) all other methods did not perform well and had lower performance scores than our model in all six metrics except PPV and F1 score (**Figure 3B**). PPV and F1 scores were lower than our RF when we used <300 HVGs for other methods. This means, despite only using the info from 167 genes, our RF model showed comparable results as five commonly used transfer methods and even better predictions when many HVGs are used for the classification.

We also evaluated all methods on a single cell expression dataset generated from FACS-sorted immune cells on Zheng dataset (27). The dataset consists of nine immune cell populations: B cells, CD14 monocytes, naïve CD8^+^ T cells, cytotoxic CD8^+^ T cells, NK cells, memory CD4^+^ T cells, naïve CD4^+^ T cells, regulatory CD4^+^ T cells and helper CD4^+^ T cells. For the comparison, we downsampled 2k cells from each cell type and used the same set-up as previously described. Our model achieved high prediction scores (scores range in 94-99) (**Figure 3A**). CHETAH (scores range in 68-93) and singleR (scores range in 87-97) again had lower prediction scores than our RF model. Our RF model had comparable results to other algorithms (scores range in 95-98, 92-99 and 95-98 for Seurat, scType and CellTypist, respectively). Like for the Kotliarov benchmarking dataset, Seurat, CellTypist and scType trained with small sets of genes (<500) performed worse than our model in all six metrics (**Figure 3B**). So, our random forest approach based on a comparably small set of genes (~170) obtained from our high-quality signature collection matched the performance or even outperformed the most widely used cell type annotation procedures (when executed with small number of HVGs) in both, the Kotliarov dataset (in which cell types had been annotated based on surface protein expression) and the Zheng dataset (in which cells had been sorted by FACS).

### Utilizing less but more robust immune cell type-specific genes for cell type classification reduces bias in downstream analysis

We further investigated the advantage of using a reduced gene set for cell typing which inherently minimizes the influence of regulated -and thereby variable- genes on cell type assignments as for individual cells. The utilization of HVGs in cell type classification in tools like Seurat, scType, and CellTypist can introduce bias into downstream statistical analyses that can undermine the accuracy of the results. Bias leading to inflated p values can possibly be introduced in different ways (see **Supplementary Figure 3** as an example how bias might be introduced). Briefly, in perturbation experiments on complex mixtures of single cells, the selection of HVGs for classification may favor the over-assignment of cells to the specific cell type that exhibits the strongest response to the stimulus. The erroneous over-assignment of cells to this specific cell type might lead to a larger sample size for statistical testing, and consequently to inflated p values.

**Figure 4:**
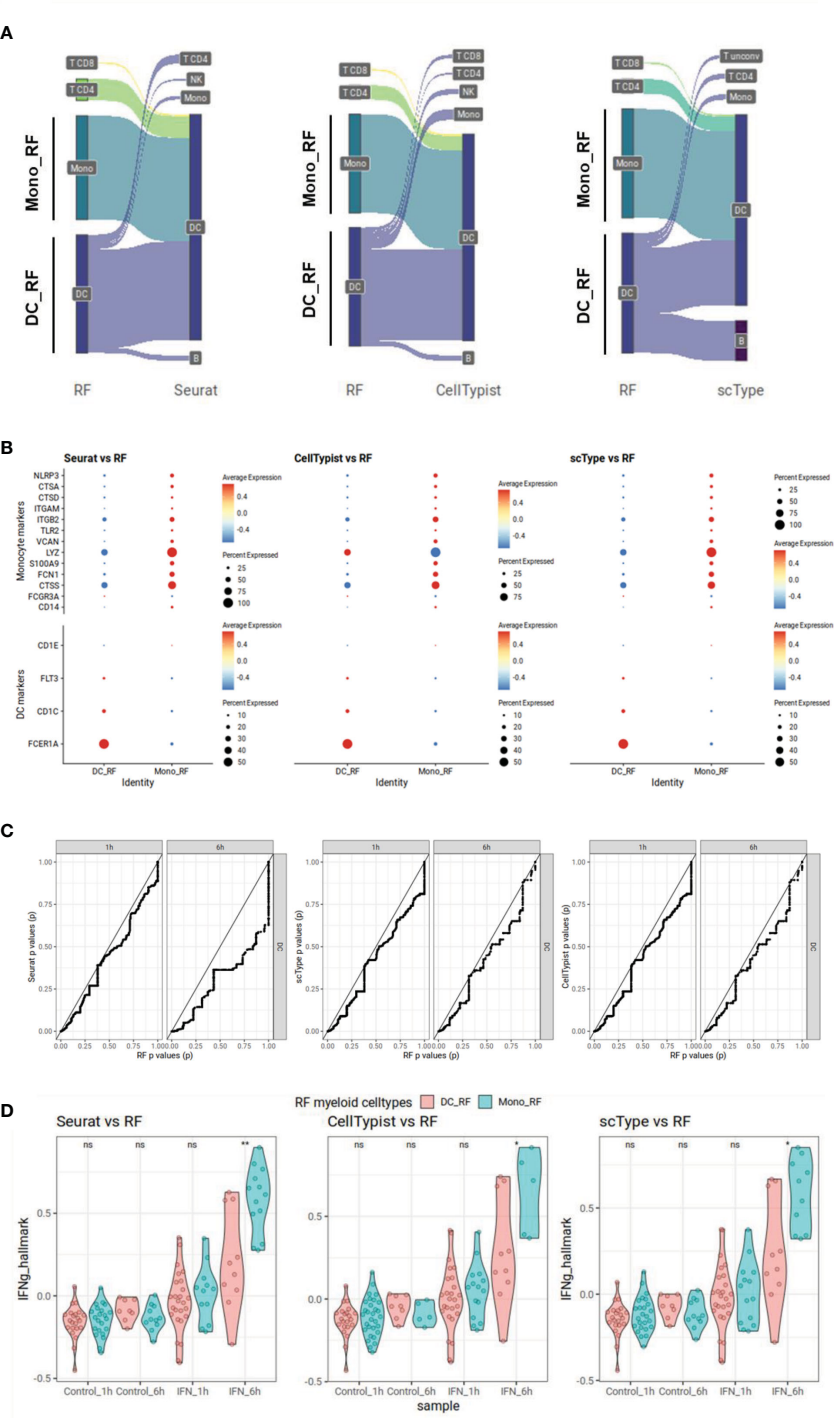
Possible downstream statistical analysis bias demonstrated in interferon gamma stimulated PBMC Kartha scRNA-seq dataset. Cell types are labeled using our random forest (RF) model utilizing our immune cell type genes, or using Seurat, scType and CellTypist. Mono_RF cells are cells labeled as monocytes in RF but as dendritic cells (DCs) in other methods while DC_RF cells are DCs labeled as DCs in RF. **(A)** Sankey plot showing different cell type assignments in RF and other methods for cells classified as DCs either by RF or other methods. **(B)** Dot plots showing expression of monocyte or DC gene markers in Mono_RF and DC_RF cells. The size of the dots represents the percentage of expression while Z-scaled average expressions are shown from blue (low) to red (high). **(C)** P-value-to-p-value scatter plots showing over-optimistic p values for Seurat, scType and CellTypist compared to RF. We perform differential gene expression analyses for 2k HVGs using the cell types defined as DCs in different cell typing methods, for each sampling time point separately. P values generated for RF-generated DC cell groups are displayed on the x-axis while DC groups from other cell typing methods are shown on the y-axis. In each comparison, the comparison line falls below the trend line pointing out over-optimistic results from other methods compared to RF. **(D)** IFNg Hallmark scores for DCs annotated by RF DC_RF or misclassified monocytes Mono_RF. For each condition, mean signature scores for IFNg Hallmark genes are calculated. DCs annotated by RF are compared with those cells classified as monocytes by RF but as DCs by other approaches. We apply Wilcoxon rank sum tests to compare IFNg Hallmark scores between those two cell type groups at each time point separately (ns = non-significant (p > 0.05); * = p < 0.05, ** = p < 0.01).

To demonstrate the potential bias introduced by using a large number of genes for cell type classification, we focused on the well-characterized effect of type II interferon (IFN-gamma, IFNg) on monocytes and dendritic cells (DCs) (42). It is known that IFNg response is strongly affecting gene expression in those myeloid cell populations. We examined the Kartha dataset (28) in which PBMCs are treated with IFNg and samples were taken at two different time points, one and six hours after stimulation. We classified cell types using our RF approach, and using Seurat, scType and CellTypist that depend on many HVGs for supervised or unsupervised classification. We conducted a comparison between the assignments of DCs when using our RF approach and those from other three methods. We observed that our RF method assigned much less DCs (n=66) compared to other methods (Seurat: n=126, scType: n=105 and CellTypist: n=115) (**Figure 4A**). Most cells classified as DCs by other three methods were classified as monocytes and DCs in RF. To further examine the monocyte-DC cell type assignment differences, we compared the cells classified as DCs by RF (DC_RF) against cells classified as monocytes in RF but as DCs in other methods (Mono_RF) with regard to the expression of known cell type markers. Mono_RF cells indeed showed lower DC markers (*FCER1A*, *CD1C*, *FLT3*, and *CD1E*) and higher monocyte markers (*CD14*, *FCGR3A*, *CTSS*, *FCN1*, *S100A9*, *LYZ*, *VCAN*, *TLR2*, *ITGB2*, *ITGAM*, *CTSD*, *CTSA*, and *NLRP3*) compared to DC_RF cells suggesting a possible misclassification of these monocytes as DCs by other three methods (**Figure 4B**). To further delineate the effect of this misclassification on potential bias in downstream analyses, we examined the results of statistical tests (Wilcoxon rank sum test) for differential gene expression (control vs. IFNg) of 2k HVGs on DCs assigned by each method. In scatter plots of p values of our approach versus p values of the other approaches, we observed that the curves consistently fell below the diagonal, a result that was especially pronounced for gene expression at 6 hours (**Figure 4C**). This suggests that other methods may produce overly optimistic p value results. To better characterize the reason for this bias, we compared IFNg responsiveness between Mono_RF (misclassified as DCs by other approaches) and DC_RF (true DCs labeled by RF). We calculated the mean signature scores for IFNg genes from IFNg Hallmark signature (43) for each cell comparing RF results with other three approaches (**Figure 4D**). In all comparisons, Mono_RF had significantly higher IFNg scores especially at 6 hours and marginally higher, yet insignificant IFNg scores after 1 hour. These results provide additional evidence supporting the notion that the misclassification of monocytes as DCs by Seurat, scType, and CellTypist contributes to inflated p values, and suggests as a reason a potential overcalling of DCs due to the expression of IFNg-related genes (among HVGs) in the misclassified monocytes.

### Using our immune cell type genes yields higher prediction than using other published immune cell type signature repertories

Finally, we assessed how our gene sets performed in RF classification approach in comparison with four other published immune cell type signatures (hereafter referred to as the Angelova, Abbas, Charoentong and Nieto gene sets) (7, 9, 11, 24). We trained all RF classifiers on subsets of genes (as defined by our or the four published signature sets) of the Hao reference dataset. We benchmarked the prediction results on scRNA-seq datasets of Kotliarov and Zheng benchmarking datasets. The comparator gene sets have been originally derived or previously utilized in different ways. The Nieto gene sets have been manually curated and have been applied on multiple scRNA-seq tumor microenvironment datasets; other three gene sets have been derived from bulk RNA-seq or microarray datasets. All four gene sets have been applied to study the tumor microenvironment. To highlight the lower bound of expected gene set performance we also assembled random sets of genes with the same number of genes which we had in our gene set repertoire (167 and 163 genes for Kotliarov and Zheng datasets, respectively).

**Figure 5:**
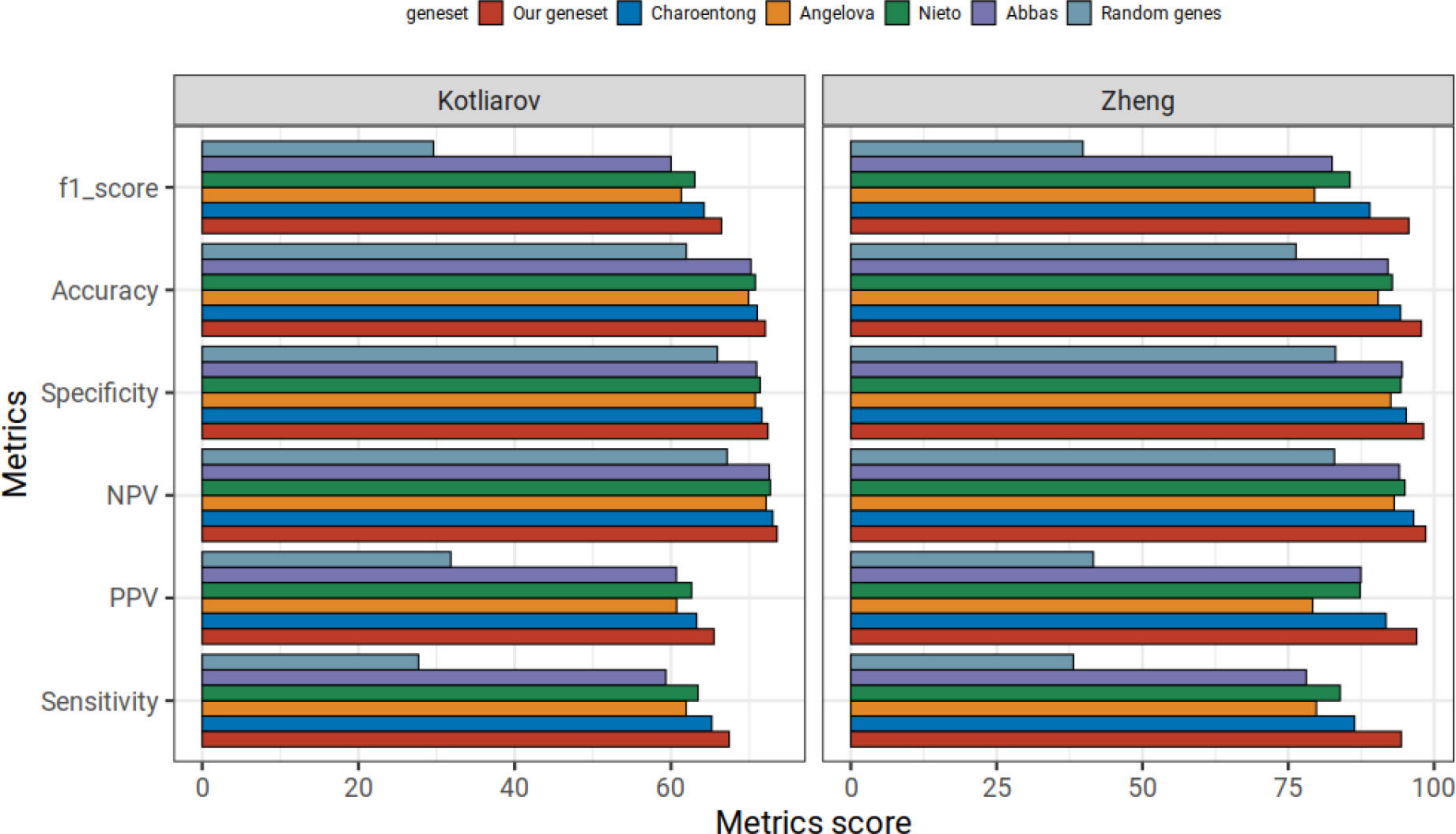
Comparison of our immune cell type signature repertoire with other published signatures on random forest approach in benchmarking datasets. Mean statistic metrics are shown for random forest models trained using our immune cell gene set and different published immune cell type repertoires (Abbas, Angelova, Charoentong and Nieto). We also include random forest models trained on random genes (with the same number of random genes as we have in our immune cell gene signature repertoire). Using our gene signatures yields better prediction performance in the benchmarking data from Kotliarov and Zheng than all approaches.

The benchmarking results for all gene sets in our random forest classification approach, i.e., the overall prediction accuracy, F1 score, sensitivity, specificity, NPV and PPV are listed in **Figure 5**. As expected, random genes showed the worst prediction scores in every metric: all gene sets clearly outperformed the randomly selected gene sets. Overall, the most favorable prediction results in every metric were obtained with our immune cell gene sets, followed by the Charoentong gene sets. In Kotliarov and Zheng datasets, the minimum difference between our gene signature to Charoentong gene set was in NPV scores (0.6 and 2.1, respectively). Conversely, we observed maximum differences to the Charoentong gene set (2.4) in the Kotliarov dataset with regard to sensitivity, PPV and F1 scores while in the Zheng dataset sensitivity scores from Charoentong had the highest difference of 8 to our gene set. In summary, for both benchmarking datasets our random forest model trained on our comparably small sets of robust immune cell type marker genes overall showed superior performance when compared to the classifiers trained on four published gene repertoires.

## Discussion and conclusions

One main objective of our study was to discover and validate robust gene expression signatures for immune cell populations based on multiple single cell datasets. To do so, we established a novel workflow consisting of dataset integration, dimensionality reduction, density-based clustering, and cluster refinement methods. In our workflow we solely analyzed similarities between gene profiles across cells. In contrast to other approaches, we avoided selecting genes based on differential expression between cell type clusters, thus avoiding problems related to masking signals of small but important expression programs when focusing on the sample (here cell) level and not the gene level (44). Our benchmarking results show that our approach can yield reasonably sized sets of marker genes with equal or better classification potential compared with published gene sets. Through application of our workflow on integrated single cell data from six studies we identified gene signatures for ten distinct immune cell type populations. We detected a relatively low gene overlap (0.0-0.3 maximum Jaccard indices, 0.08-0.67 for maximum S-S indices) between our signatures and published signatures. This confirms the novelty of our approach but is also not astonishing since each published gene signature had been derived using different analytical methods, different sequencing technologies, or stems from investigations of different cell populations. Like our results about gene set overlap, Nirmal et al. reported low concordance between their signatures and other published signatures (32). Interestingly, their B cell signature when compared to our B cell signature yielded the highest Jaccard and SS indices. Low Jaccard scores to published signatures highlight the difficulty of the problem to identify robust signatures on the one hand, and the novelty of our approach to derive signatures on the other hand. Finally, our immune cell gene expression signatures yield better cell type classification results compared to other published signatures. So, we argue that they are a reasonable choice as feature sets for cell type classification.

Another aim of our study was to identify cell type classification approaches that might be competitive when compared to existing methods for cell typing in complex scRNA-seq datasets. To this end, we developed a random forest classifier trained only on expression data for our gene sets. We compared our predictions with five published cell type annotation algorithms. These algorithms were shown by others to perform well in different benchmarking studies: especially Seurat was gaining attention and currently is probably the most widely used cell type annotation tool (45). On our PBMC benchmarking datasets, the Kotliarov and Zheng datasets, we showed that our random forest model trained only using our gene signature collection achieved comparable prediction scores that are always close to the best performing approach of Seurat, scType and CellTypist. However, an important difference between these cell typing methods and our method is that these approaches use mostly 10-fold more genes (2000 HVGs instead of ~170 distinct signature genes) for cell typing. For downstream statistical analyses we therefore leave >90% of HVGs and their data untouched by classification. This means that those HVGs can be subjected to downstream differential gene expression testing without bias. Such bias can arise when the same gene profiles are first used to establish cell type information (for example through clustering-based cell tagging) and later are tested for differential expression between groups that have already been defined using the same profiles. This is especially important for more complex experimental designs since most cell typing approaches face the problem of downstream analysis bias. The selection of HVGs might compromise those genes that are affected by important experimental variables such as a treatment stimulus and time point. We could clearly show how statistical bias could be introduced for an experiment with type II interferon treated DCs and monocytes (**Figure 4**). In this well characterized stimulation experiment, the p values obtained with cell type labeled using Seurat, scType and CellTypist led to an inflation of significance in the p values from 2k HVGs. We show that one reason for the bias is the inappropriate labeling of non-DC cells, that respond to the interferon stimulus, leading to larger numbers of cells with DC labels, thereby increasing the sample size for “DC cells” and leading to overoptimistic p values for many interferon-regulated genes. Especially for such a stimulation experiment, it is important that results from downstream statistical analyses of the most variable genes are not biased. Currently, most single cell datasets do not have multiple experimental variables. In the future, single cell experimental set-ups could become more complex, especially when complex perturbations or time courses are to be analyzed. Then, providing cell types in a way that allows unbiased downstream analysis will become increasingly important.

Some reports suggest that there are considerable differences in gene expression profiles of immune cells in different cellular environments, and that this leads to poor performance when an approach for cell typing is developed in one tissue context and then used in another tissue context (32, 46, 47). In our approach, we did not perceive tissue switching as a problem. We obtained our immune cell type signatures by leveraging multiple tumor microenvironment and tested their utility in cell type classification on two benchmarking PBMC datasets with medium to excellent performance (scores in range 94-99 in Zheng and 65-72 in Kotliarov - high score in Kotliarov despite two cell type populations being not included in the reference dataset).

In summary, our workflow is a valid approach to discover novel robust gene sets based on gene similarities from multiple scRNA-seq datasets. We demonstrated the superior performance of our immune cell type signatures compared to other gene sets in a random forest cell typing approach on two benchmarking datasets. In addition, we showed that random forest classifiers that use our gene sets as features match the performance of most widely used approaches for cell typing in scRNA-seq data, even though they do not make use of expression from many selected highly variable genes. For these untouched genes this generates opportunities for unbiased downstream statistical analyses making use of cell type information. Our results make us confident that our immune cell type signatures combined with a random forest approach can be used to analyze further complex single cell data. With even more data becoming available in a quickly growing universe of scRNA-seq gene expression data we are sure that our signature discovery approach can lead to many further cell type signature discoveries, also in other tissues than the tumor microenvironment or PBMCs.

## Data availability statement

The original contributions presented in the study are included in the article/**Supplementary Material**. Further inquiries can be directed to the corresponding author. The scripts and outputs from this study will be deposited on https://github.com/ba306/immune-cell-signature-discovery-classification-paper.

## Ethics statement

The studies involving human participants were reviewed and approved by the original authors for the informed consent in publicly available datasets. The patients/participants provided their written informed consent to participate in this study.

## Author contributions

ES: conceptualization, methodology, supervision, funding acquisition, reviewing, and editing. BA: conceptualization, methodology, software, visualization, data curation, formal analysis, investigation, writing, original draft preparation, reviewing, and editing. SZ: data curation and supervision; BB: supervision and reviewing. All authors contributed to the article and approved the submitted version.
