# Supplementary material for: Immune cell type signature discovery and random forest classification for analysis of single cell gene expression datasets
Source: Front Immunol. 2023 Aug 4;14:1194745. doi: 10.3389/fimmu.2023.1194745 (PMC10441575; doi:10.3389/fimmu.2023.1194745)
Supplement: Supplementary file 1 [file DataSheet_1.zip › Supp_Fig_Tab/Supplementary Fig legends.docx]

# Supplementary Figures

**Supplementary Figure 1. Violin plots for validating the gene signatures in the discovery and validation datasets.** Mean signature scores are calculated for each signature and each cell in discovery and validation datasets. High expression of a gene set in the corresponding cell type is tested using Wilcoxon rank sum tests (ns = non-significant (p > 0.05); * = p<0.05, ** = p<0.01).

**Supplementary Figure 2. Heatmap of Jaccard index scores (A) and Szymkiewicz–Simpson coefficients (B) between our immune cell type signatures and seven other published immune cell type signatures.** Jaccard index scores and Szymkiewicz–Simpson coefficients are calculated between our eleven refined gene signatures (rows) and seven published cell signatures (columns). The number of genes in each gene set has been indicated inside brackets.


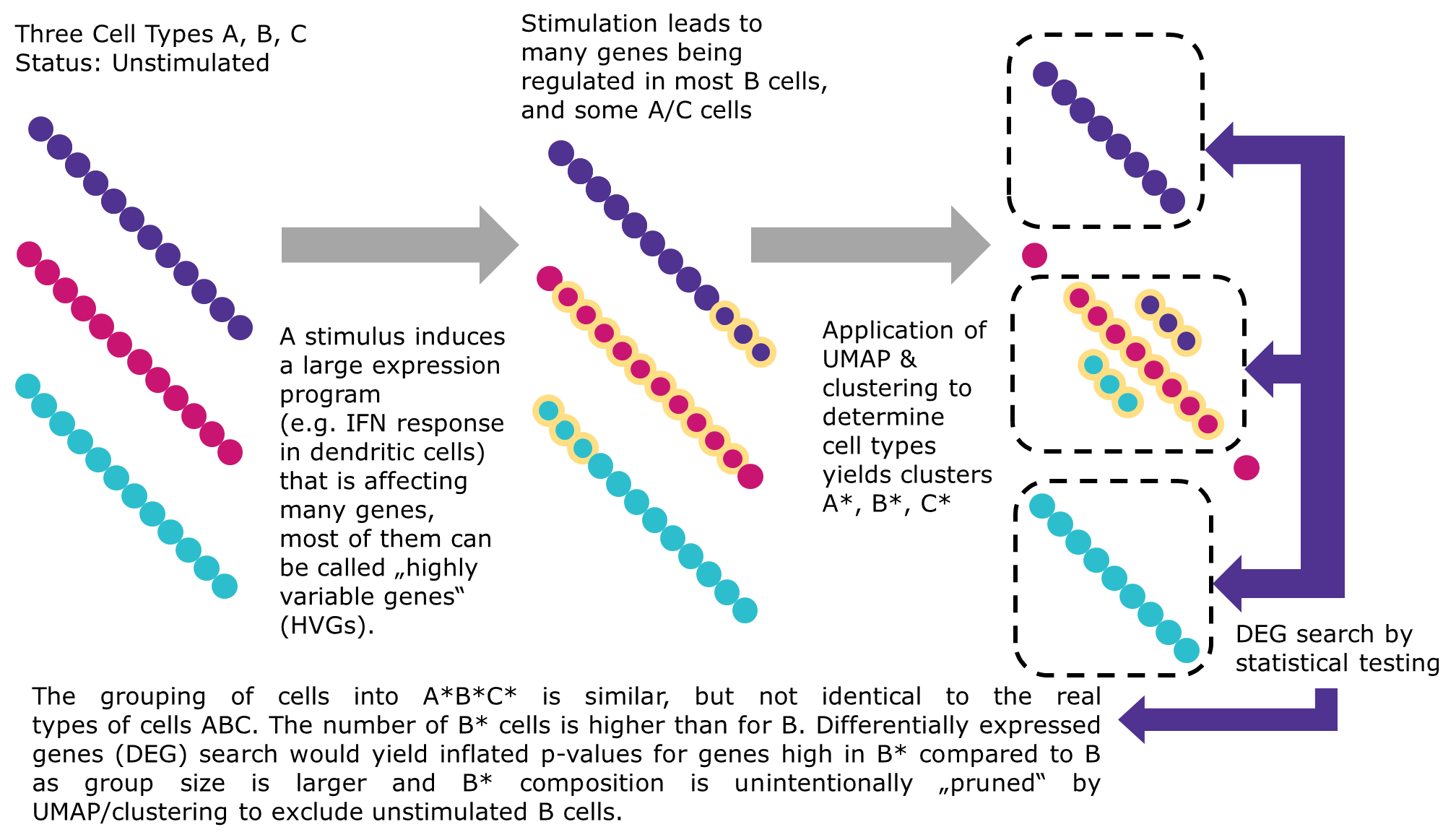


**Supplementary Figure 3. Explanation schema for the possible downstream analysis bias.**
