# Supplementary material for: Immune cell type signature discovery and random forest classification for analysis of single cell gene expression datasets
Source: Front Immunol. 2023 Aug 4;14:1194745. doi: 10.3389/fimmu.2023.1194745 (PMC10441575; doi:10.3389/fimmu.2023.1194745)
Supplement: Supplementary file 1 [file DataSheet_1.zip › Supp_Fig_Tab/Supplementary Tables.docx]

**Supplementary Table 1. Medium-depth level cell type harmonization.** For benchmarking and reference datasets cell types are summarized into medium-level categories. Larger groups are in bold and specific cell types belonging to those groups in each dataset are listed. ‘Other cells’ category from the reference dataset are removed from the training.

| **Groups** | | **Benchmarking datasets** | | | | **Reference dataset** | | |
| --- | --- | --- | --- | --- | --- | --- | --- | --- |
|  |  | **Kotliarov** | | **Zheng** |  | **Hao** | |  |
| **B** | | B transitional  B switched  B unswitched |  | B | | B memory  B naive  B intermediate  Plasma | |  |
| **Monocyte** | | CD14 monocyte  CD16 monocyte |  | CD16 monocyte | | CD14 monocyte  CD16 monocyte | |  |
| **DC** | | DC  pDC | | - |  | DC  pDC | |  |
| **NK** | | NK | | NK | | NK |  |  |
|  | **CD4^+^ T** | T CD4 memory  T CD4 naïve | | T CD4 naïve  T CD4 memory  T reg (T CD4 regulatory)  T CD4 helper | | T CD4 naïve  T CD4 EM (effector memory)  T CD4 CM (central memory)  T CD4 activated  T reg  T CD4 CTL (cytotoxic activity)  T CD4 proliferating | |  |
| **CD8^+^ T** | | T CD8 memory  T CD8 naïve | | T CD8 cytotoxic  T CD8 naïve |  | T CD8 naïve  T CD8 EM  T CD8 CM  T CD8 activated | |  |
| **T unconventional** | | Double negative T cells (dn T)  unconventional CD161^hi^/CD3^+^/CD8^+^ T cells | | - |  | **Other cells:**  Mucosal-associated invariant T cells, gamma delta T cells (gdT), dnT, HSC, innate lymphoid cells, erythrocytes, platelets | |  |
| **HSC** | | Hematopoietic stem cells (HSC) | | - | |  | | |
