# Supplementary figures and images for: Immune cell type signature discovery and random forest classification for analysis of single cell gene expression datasets
Source: Front Immunol. 2023 Aug 4;14:1194745. doi: 10.3389/fimmu.2023.1194745 (PMC10441575; doi:10.3389/fimmu.2023.1194745)

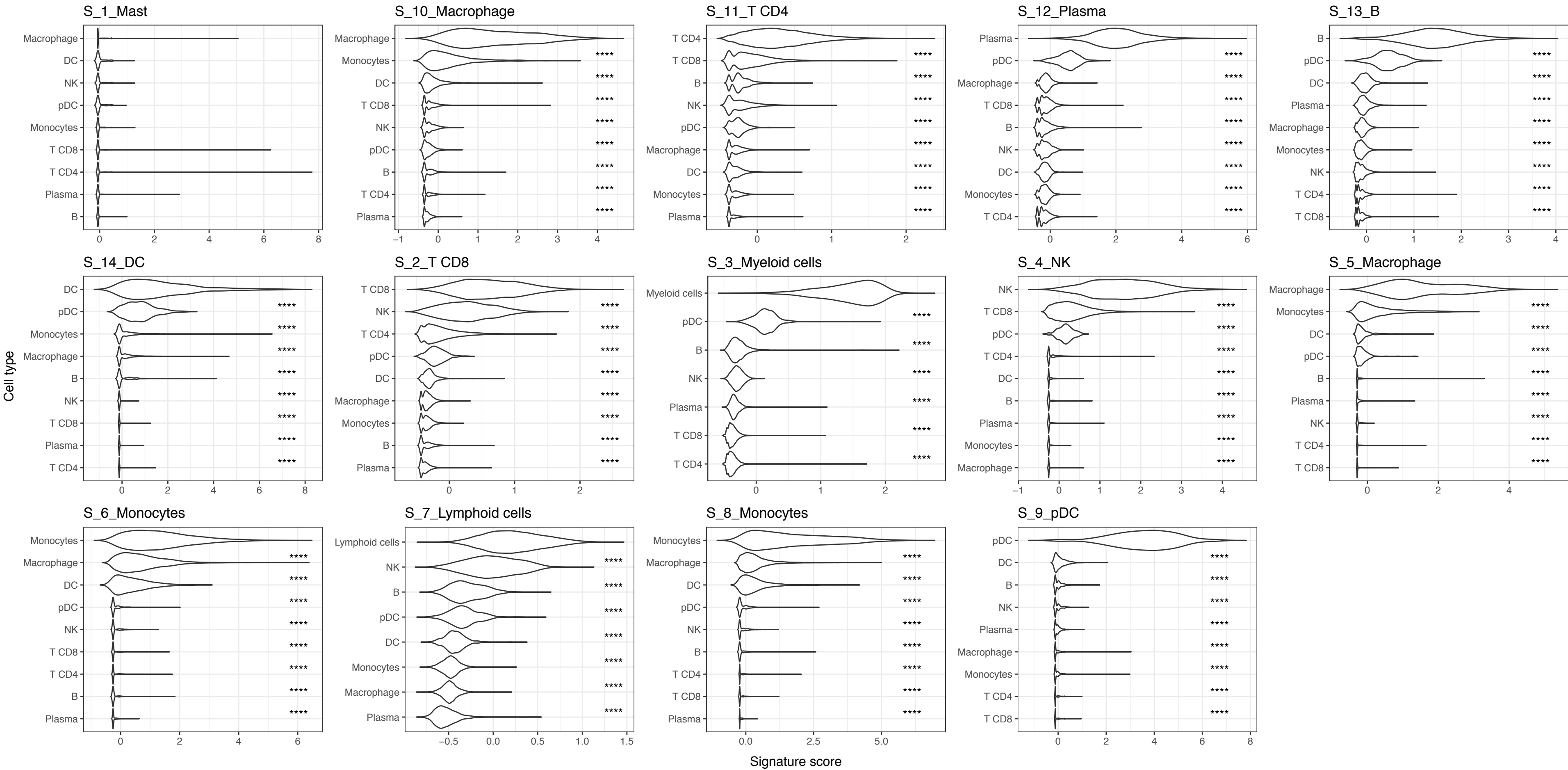

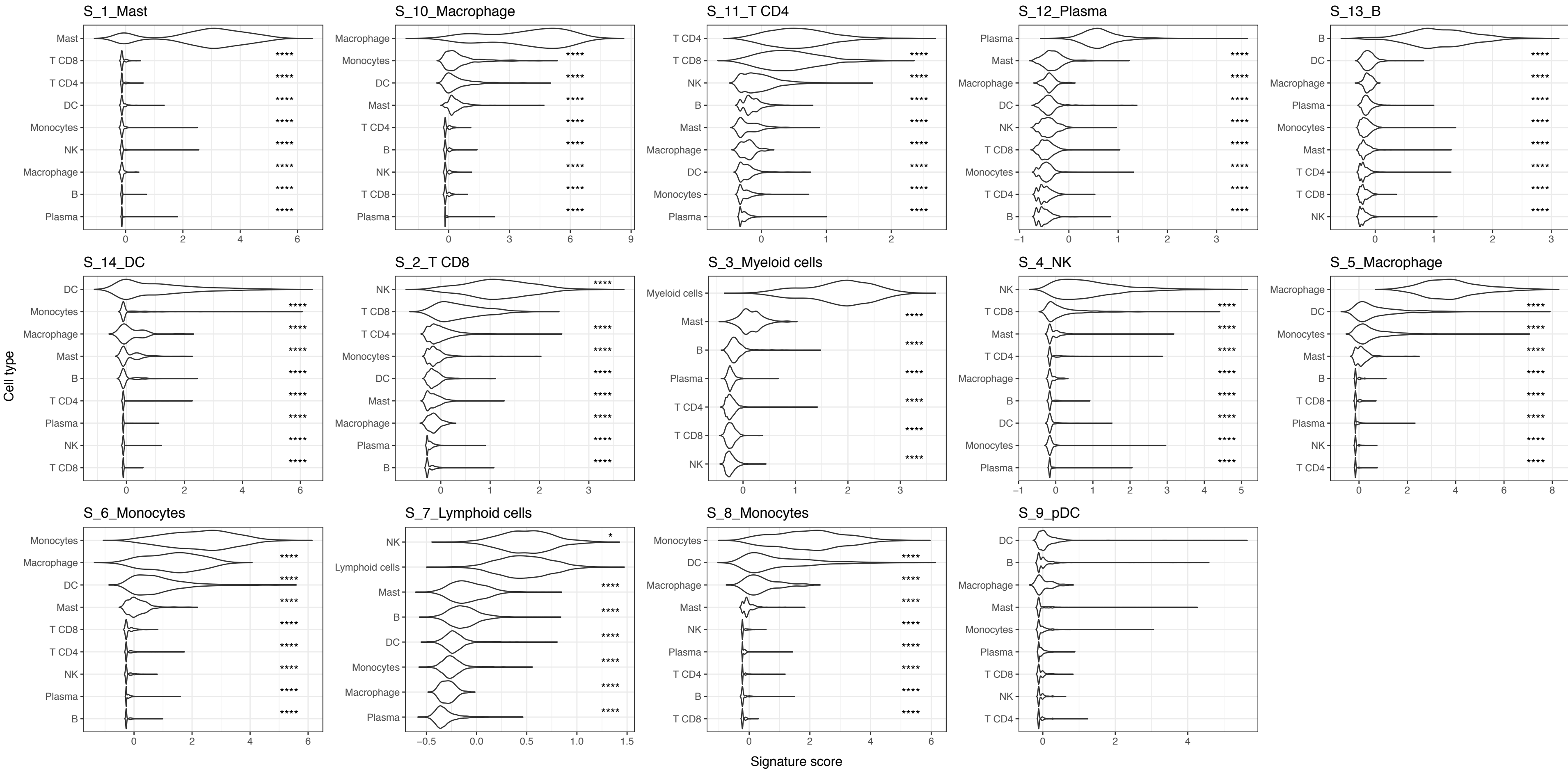

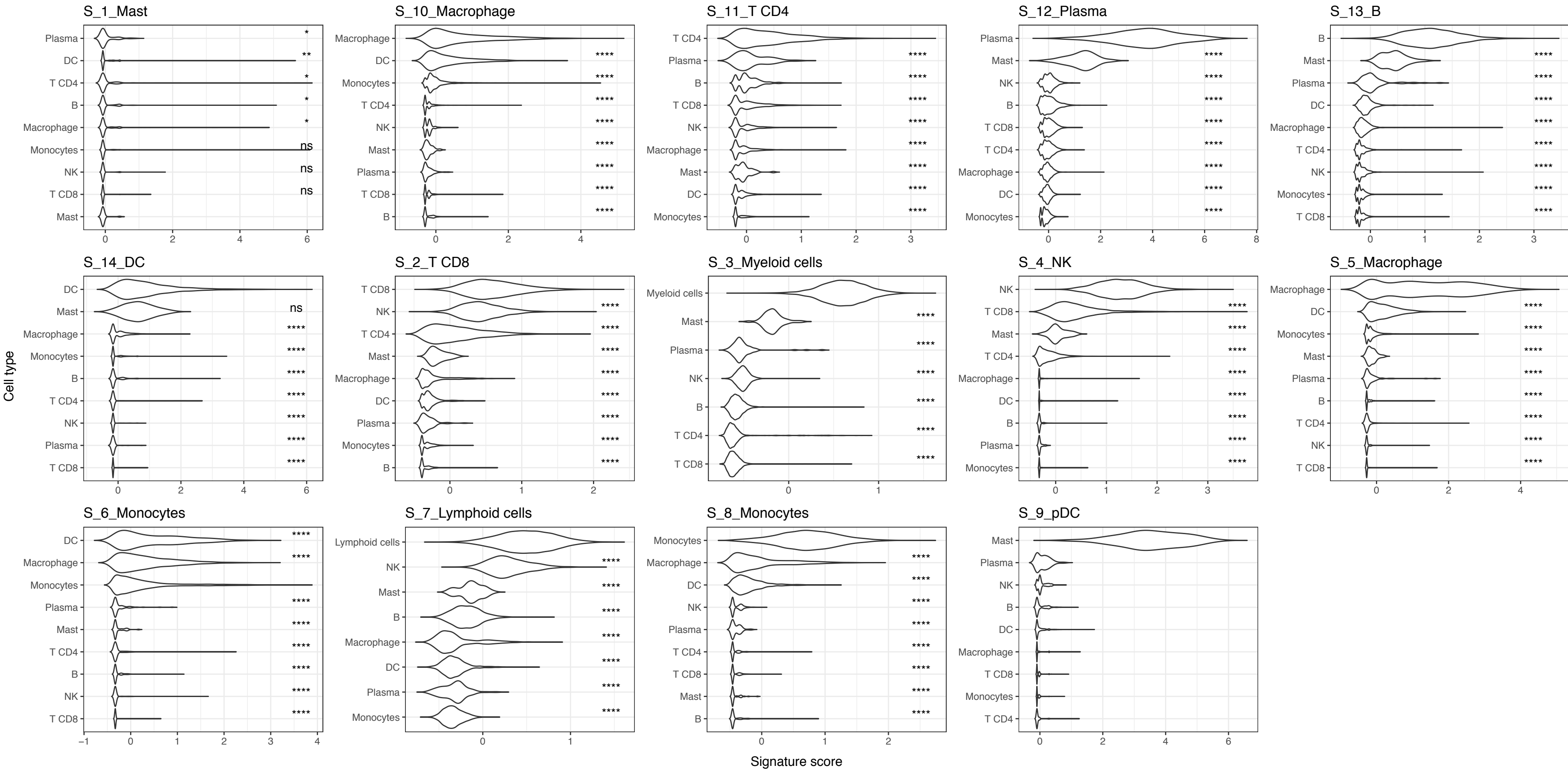

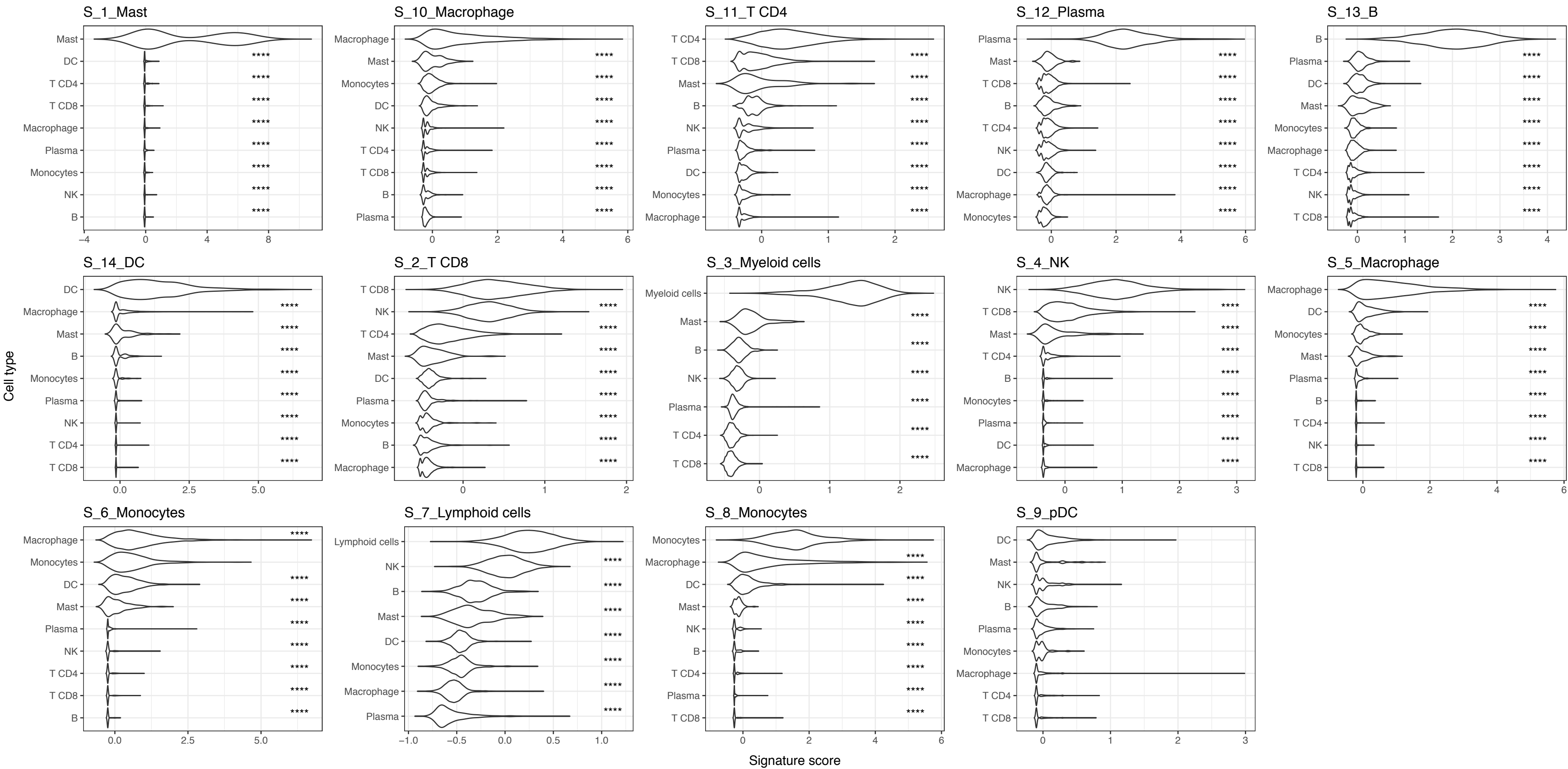

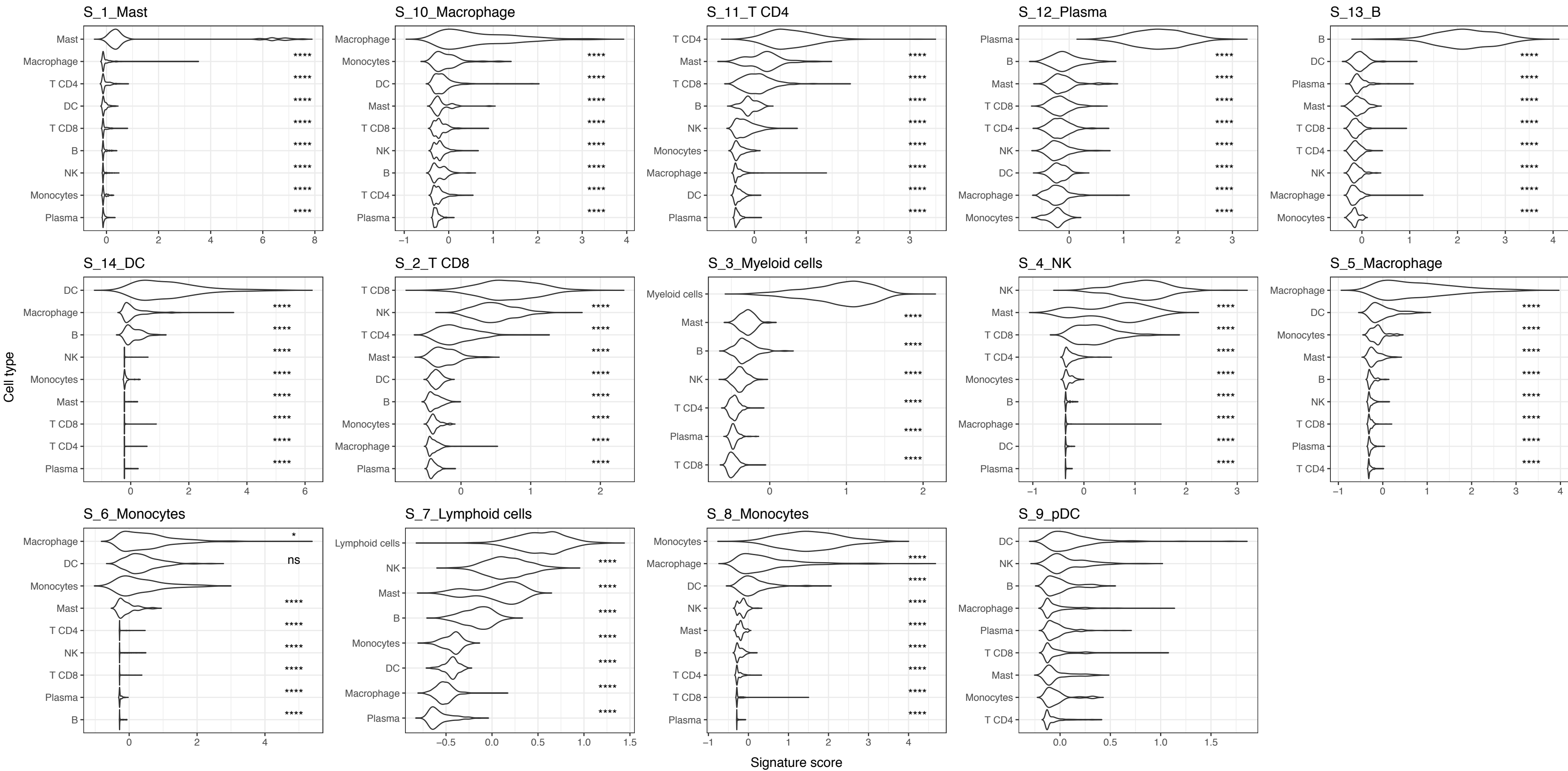

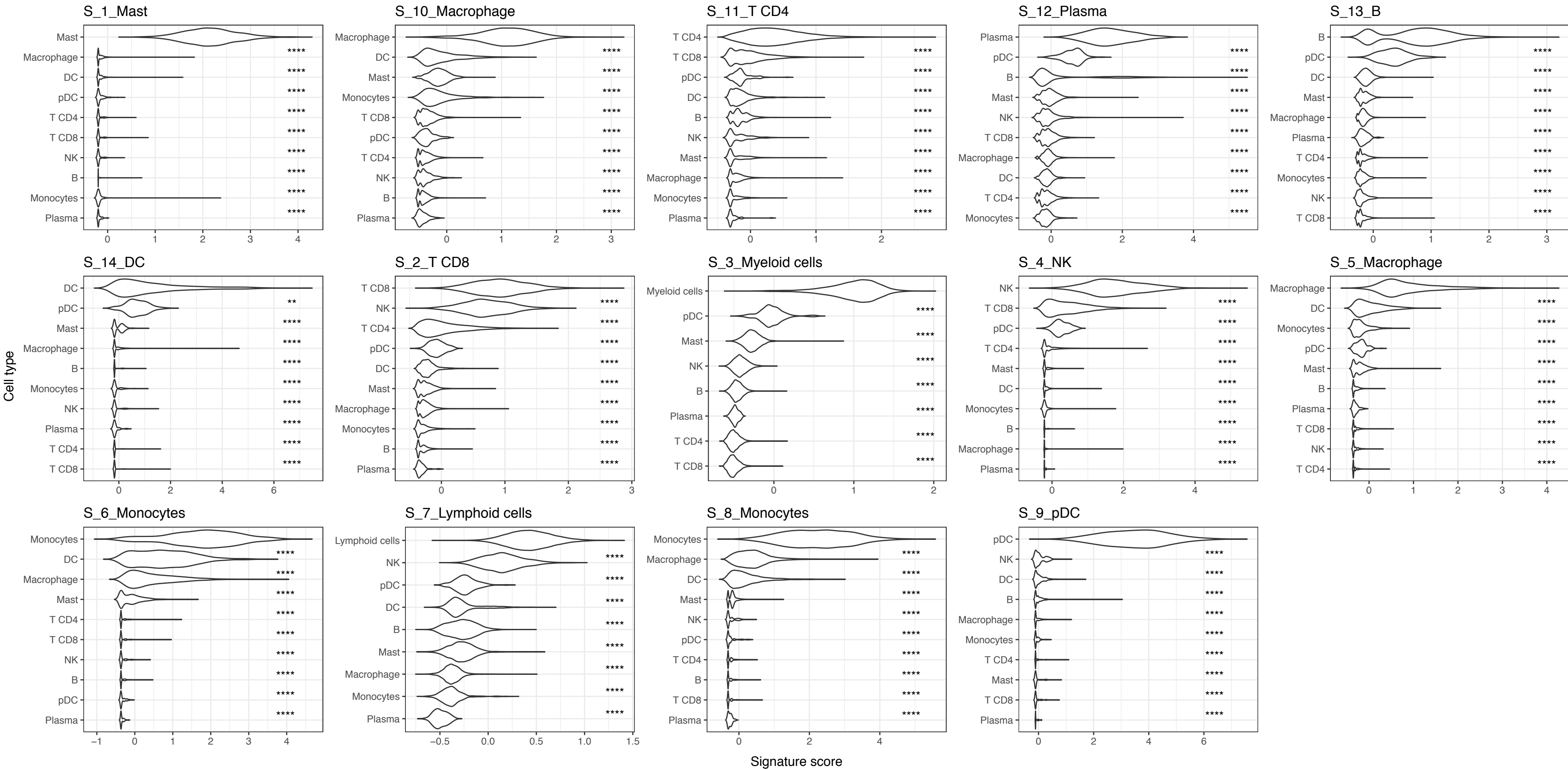

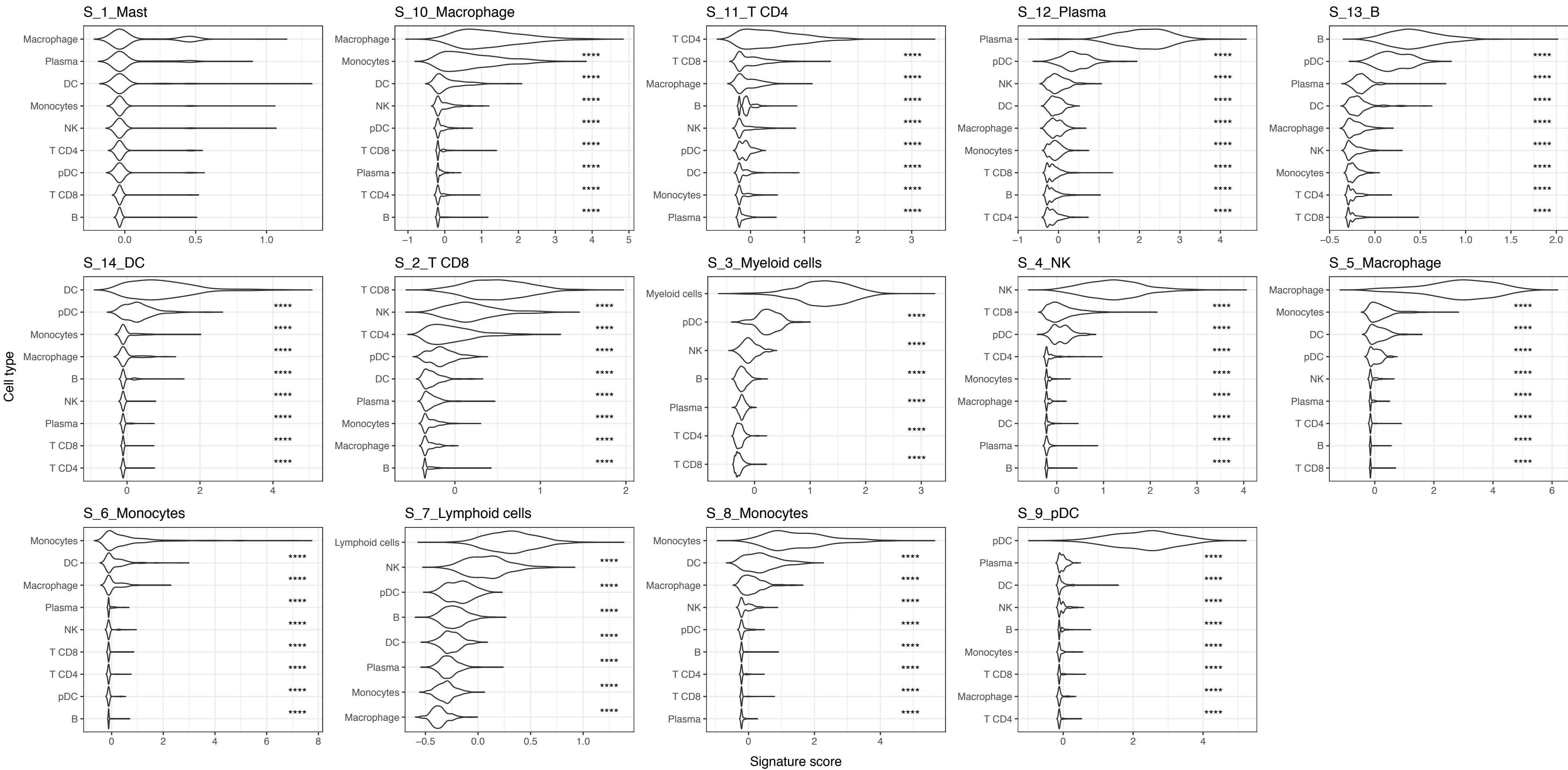

S\_1\_Mast

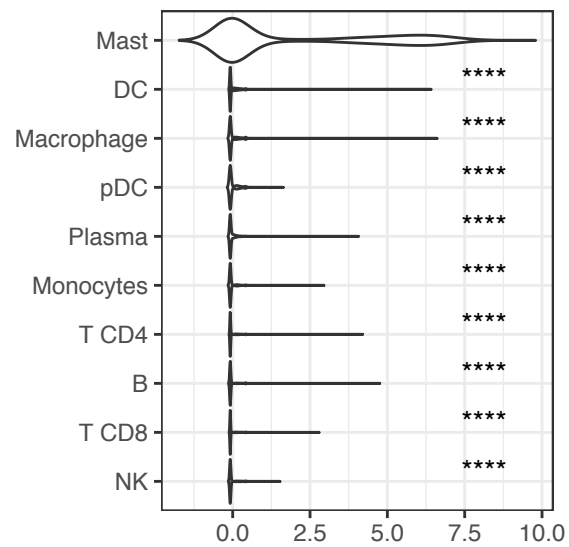

S\_10\_Macrophage

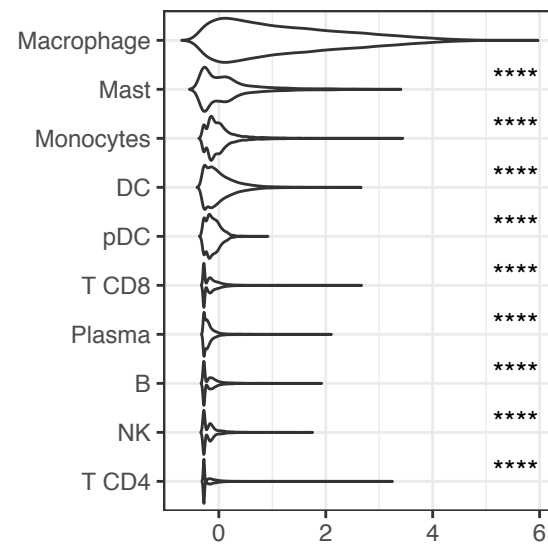

S\_11\_T CD4

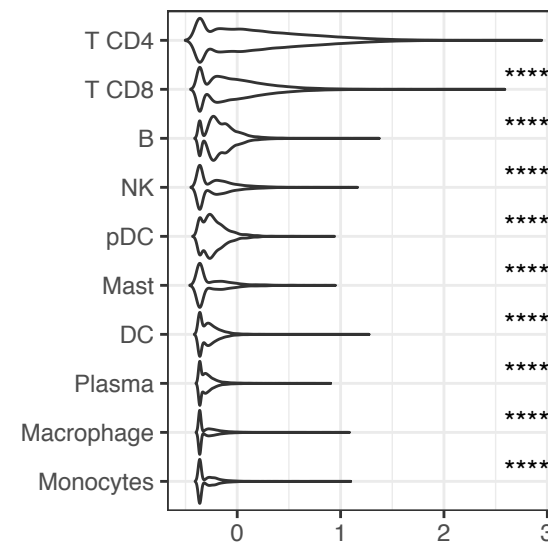

S\_12\_Plasma

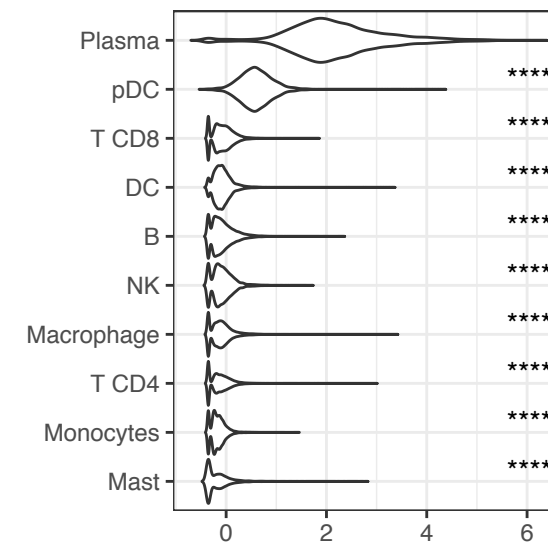

S\_13\_B

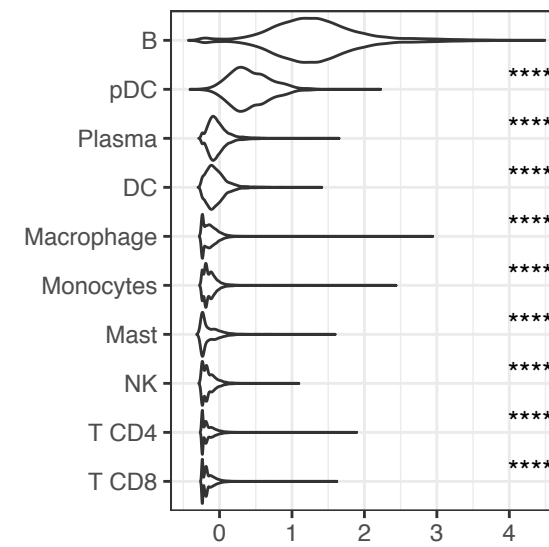

S\_14\_DC

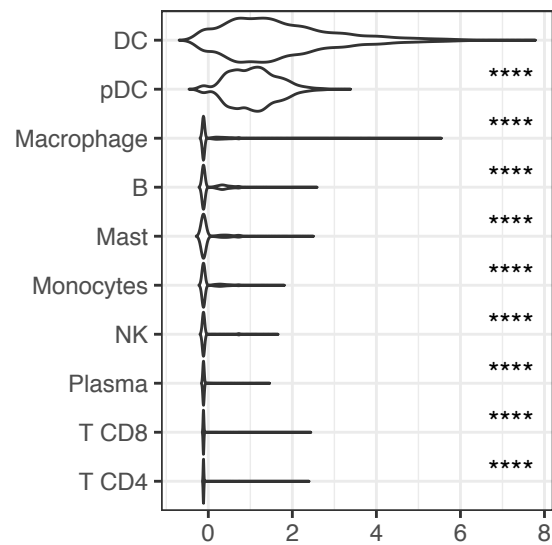

S\_2\_T CD8

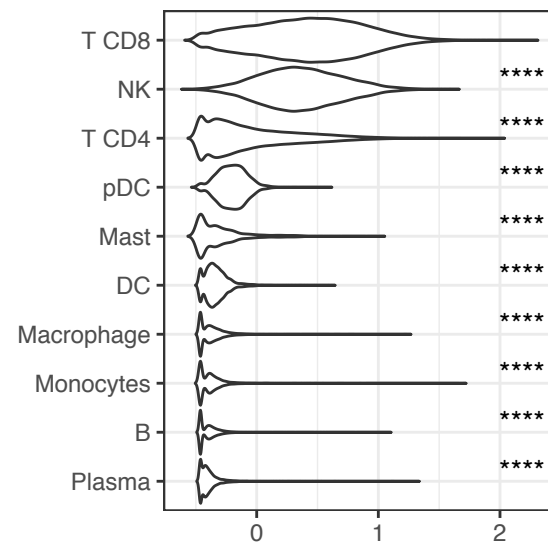

S\_4\_NK

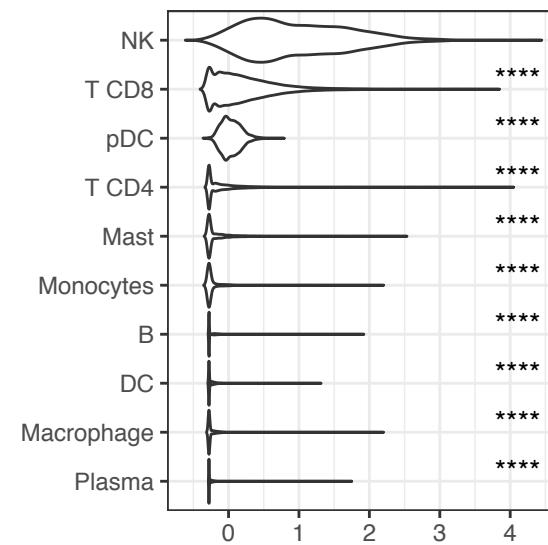

S\_5\_Macrophage

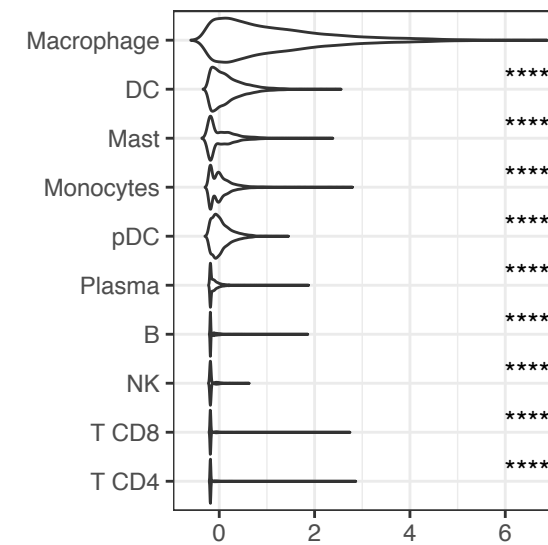

S\_6\_Monocytes

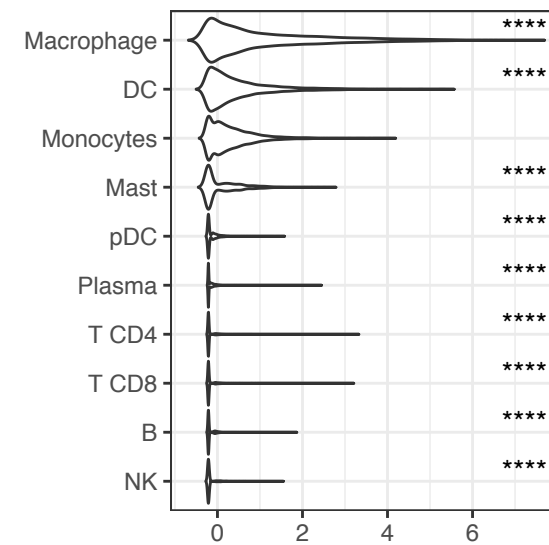

S\_8\_Monocytes

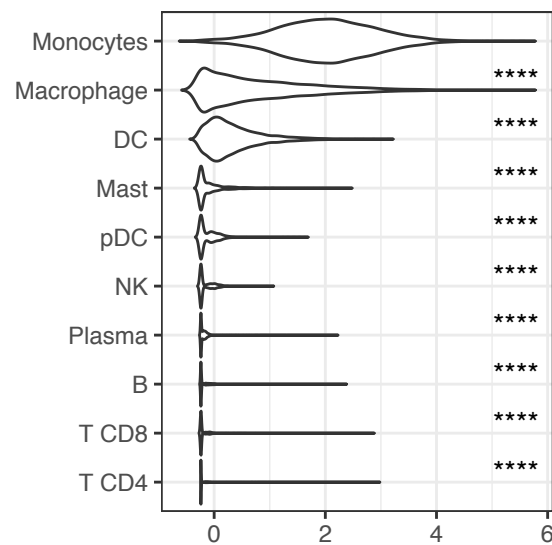

S\_9\_pDC

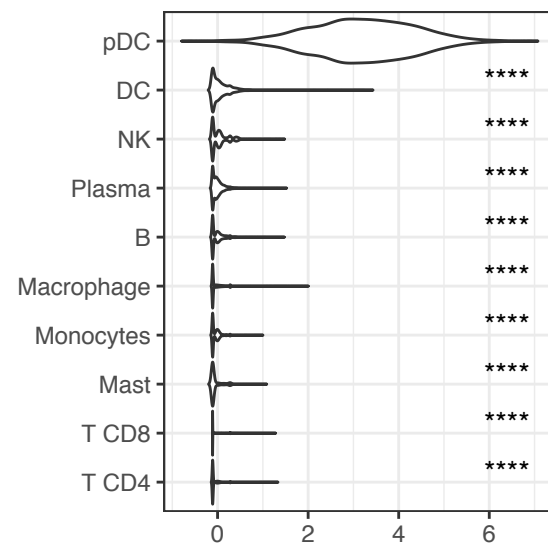

Supplement: Supplementary file 1 [file DataSheet_1.zip › Supp_Fig_Tab/SuppFig1.pdf]
